# Cyclopaldic Acid, the Main Phytotoxic Metabolite of *Diplodia cupressi*, Induces Programmed Cell Death and Autophagy in *Arabidopsis thaliana*

**DOI:** 10.3390/toxins14070474

**Published:** 2022-07-11

**Authors:** Simone Samperna, Marco Masi, Maurizio Vurro, Antonio Evidente, Mauro Marra

**Affiliations:** 1 Department of Biology, University of Rome Tor Vergata, 00133 Rome, Italy; simone.samperna@libero.it; 2 Department of Chemical Sciences, University of Naples Federico II, 80126 Naples, Italy; marco.masi@unina.it (M.M.); evidente@unina.it (A.E.); 3 Institute of Sciences of Food Production, National Research Council, 70126 Bari, Italy; maurizio.vurro@ispa.cnr.it

**Keywords:** *Seiridium cupressi*, necrotrophic fungi, phytotoxins, cyclopaldic acid, *Arabidopsis thaliana*, plant immunity, plasma membrane H^+^-ATPase, vacuole-mediated PCD, autophagy

## Abstract

Cyclopaldic acid is one of the main phytotoxic metabolites produced by fungal pathogens of the genus *Seiridium*, causal agents, among others, of the canker disease of plants of the Cupressaceae family. Previous studies showed that the metabolite can partially reproduce the symptoms of the infection and that it is toxic to different plant species, thereby proving to be a non-specific phytotoxin. Despite the remarkable biological effects of the compound, which revealed also insecticidal, fungicidal and herbicidal properties, information about its mode of action is still lacking. In this study, we investigated the effects of cyclopaldic acid in *Arabidopsis thaliana* plants and protoplasts, in order to get information about subcellular targets and mechanism of action. Results of biochemical assays showed that cyclopaldic acid induced leaf chlorosis, ion leakage, membrane-lipid peroxidation, hydrogen peroxide production, inhibited root proton extrusion in vivo and plasma membrane H^+^-ATPase activity in vitro. qRT-PCR experiments demonstrated that the toxin elicited the transcription of key regulators of the immune response to necrotrophic fungi, of hormone biosynthesis, as well as of genes involved in senescence and programmed cell death. Confocal microscopy analysis of protoplasts allowed to address the question of subcellular targets of the toxin. Cyclopaldic acid targeted the plasma membrane H^+^-ATPase, inducing depolarization of the transmembrane potential, mitochondria, disrupting the mitochondrial network and eliciting overproduction of reactive oxygen species, and vacuole, determining tonoplast disgregation and induction of vacuole-mediated programmed cell death and autophagy.

## 1. Introduction

**Figure 1:**
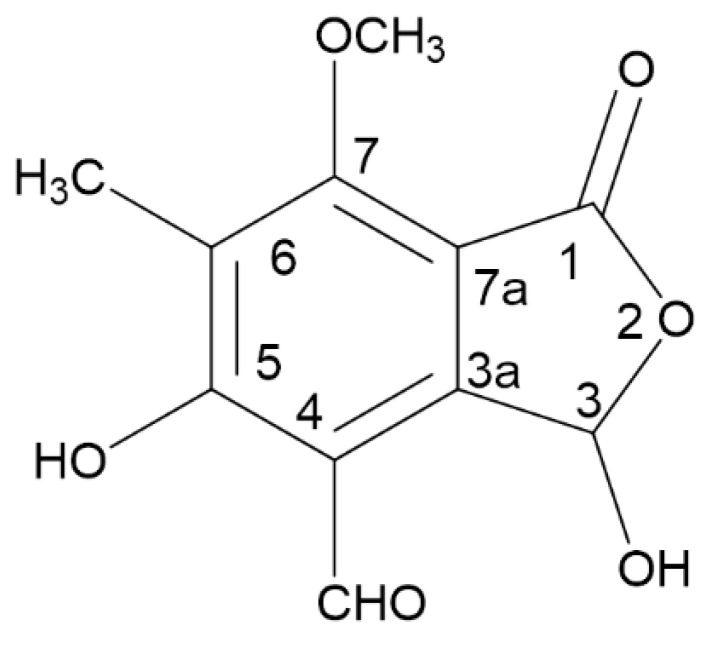
Chemical structure of cyclopaldic acid.

*Diplodia cupressi* (syn. *Seiridium cupressi*) together with *Seiridium cardinale* and *Seiridium unicorne* is the fungal causal agent of the canker disease of several plant species of the Cupressaceae family (e.g., *Cupressus* and other genera). These fungal pathogens were extensively studied between the 1970s and 1990s—being the cause of the death of millions of plants in the Mediterranean basin (particularly *Cupressus macrocarpa* and *Cupressus sempervirens*) and other ecological regions [1]. The pathogen, after penetration, produces a necrotic lesion of the bark, through which it spreads in the cortical parenchymas and in the phloem. Eventually, all tissues including the cambium turn brown and die. Cell necrosis of the cankered bark progresses steadily until the branches or stem are girdled. The appearance of symptoms caused by the infection of *Seiridium* species on their hosts, as well as the type of damage to the infected tissues (necrosis), leads us to suppose that some toxins may be produced in the cypress bark or wood colonized by the fungus. For this reason, deep investigations were carried out in order to evaluate if and which toxins could be involved in the development and severity of the disease. Each species of *Seiridium* proved to produce at least one major toxin as well as several minor phytotoxic metabolites in vitro. In particular, *S. cupressi* proved to produce two major toxins, i.e., seiricuprolide (SCU), a 14-macrolide and cyclopaldic acid (CA) (Figure 1), a pentasubstituted isobenzofuranone which was already known as an antifungal metabolite produced by some species of *Aspergillus* [2,3], *Penicillium* [4,5] and *Pestalotiopsis* sp. [6].

At concentrations 10–100 μg/mL, CA induced marked leaf chlorosis and necrosis in severed cypress twigs as well as in cuttings of non-host herbaceous plants, such as tomato, oat, and mung bean [7,8]. Moreover, subepidermal injections of the toxin into the stem of seedlings of three differently susceptible cypress species reproduced the systemic symptoms of the disease [9,10]. Furthermore, CA was accumulated in shoots of cypress seedlings inoculated with *S. cupressi* [11]. These pieces of evidence definitely demonstrated that CA is a non-specific in vivo phytotoxin. More recently, in the search for natural products as an alternative to synthetic pesticides, CA has been reported to possess insecticidal [12,13,14], fungicidal [15] as well as herbicidal [16] activities. However, despite its remarkable biological properties, information about the effects of CA at the cellular and molecular level is still very poor. In plants, CA induced loss of electrolytes from cypress shoot tissues [8], although in vitro it inhibited electron transport and oxidative phosphorylation of sweet potato and mung bean mitochondria [17] as well as esterases activity, such as lipases and acilases [8,18]. In the present study, the effects of CA administration on *Arabidopsis thaliana* seedlings or protoplasts have been investigated at the subcellular, biochemical and molecular levels, in order to shed light on the mechanism of CA phytotoxicity. Results about cell viability, electrolyte leakage, malondialdehyde (MDA) and ROS production as well as of qRT-PCR analysis of defense-related genes expression and confocal microscopy imaging of subcellular organelles are reported.

## 2. Results

### 2.1. CA Reduced Growth of A. thaliana Seedlings, Induced Leaf Chlorosis, Chlorophyll Loss and Inhibited Root Proton Extrusion

**Figure 2:**
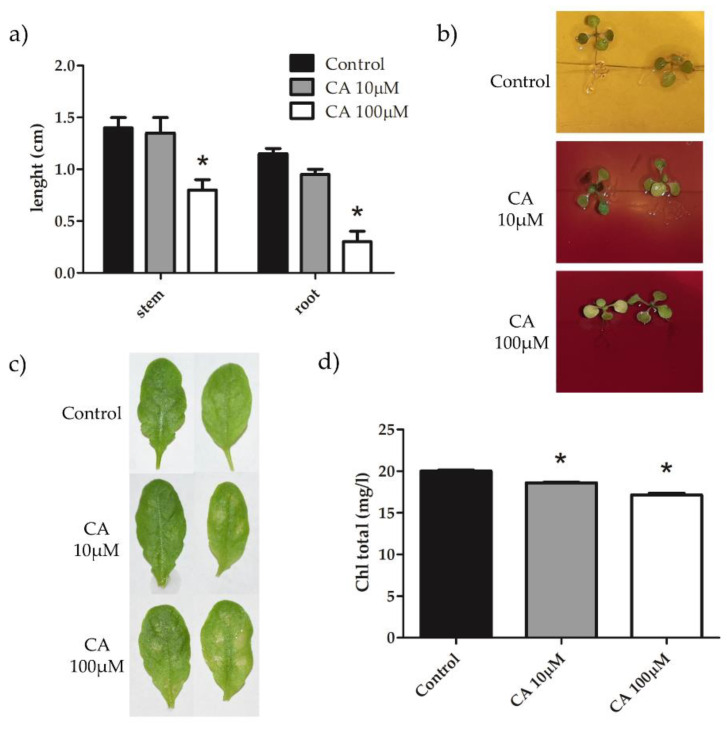
Stem and root length (**a**), root H^+^ extrusion (**b**), chlorotic areas (**c**) and chlorophyll content (**d**) of *A*. *thaliana* seedlings treated with CA. (**a**) Plants were grown in vitro in MS medium supplemented or not with 10 or 100 μM CA. Stem and root length was measured 7 dat. (**b**) Plants were grown in vitro in half-strength MS medium at pH 6, containing 0.03% Bromocresol purple. After two weeks, seedlings were treated with 10 µM or 100 µM CA and pH change monitored by observing color change 24 hat. (**c**) Leaves of three-weeks old *A. thaliana* plants were detached and inoculated with 5 μL droplets of a solution containing 10 or 100 μM CA and the formation of chlorotic areas was observed 24 hat. (**d**) Leaves of three-weeks old *A. thaliana* plants, sprayed with a solution containing 10 or 100 μM CA, were collected 3 dat and chlorophyll content was evaluated as reported in Section 5.3. Results from three independent experiments are reported; values are expressed as the mean ± SEM. Statistical significance was attributed by Student’s test (* *p* < 0.05).

The phytotoxicity of CA to *A. thaliana* plants was investigated by treating seedlings or rosette leaves with 10 or 100 μM concentrations of the toxin, according to conditions described in Section 5. CA supplemented at 100 μM into the medium significantly reduced both stem and root growth 7 days after treatment (dat) (Figure 2a). CA at 10 μM, and to a greater extent at 100 μM, hampered H^+^-extrusion in roots (Figure 2b), which is mediated by the plasma membrane H^+^-ATPase (PMA) and is a key process to provide energy for cellular transport and growth [19]. Application of 5 μL droplets (with 10 or 100 μM CA) to detached rosette leaves induced chlorosis 24 h after treatment (hat) and the area of chlorotic lesions increased together with CA concentration (Figure 2c). This result was paralleled by a similarly concentration-dependent reduction of the chlorophyll content of *A. thaliana* leaves sprayed with a solution of 10 or 100 μM CA, 3 dat (Figure 2d). These data, on the whole, were in accordance with previous observations concerning phytotoxicity of CA in other plant species [7,8] and demonstrated that *A. thaliana* is a valuable model to study the mechanism of CA phytotoxicity.

### 2.2. CA Induced Ion Leakage, Hydrogen Peroxide Production, Membrane-Lipid Peroxidation and Reduced Cell Viability in A. thaliana Leaves

**Figure 3:**
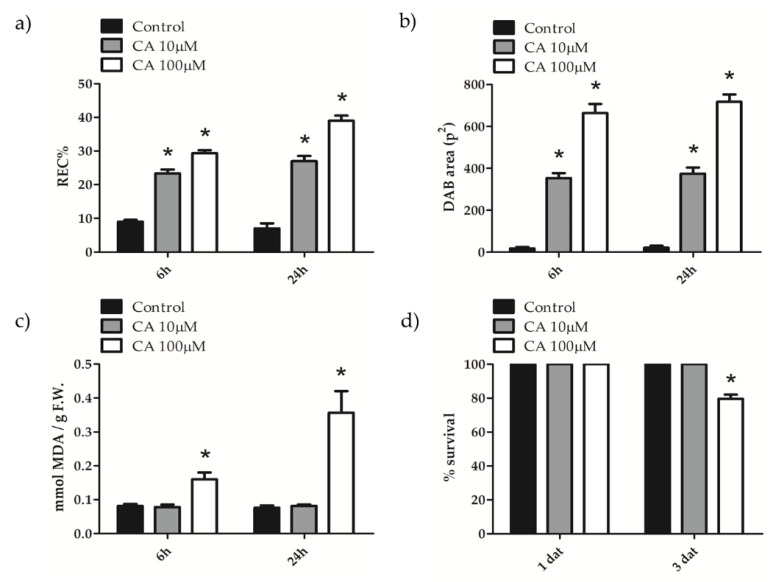
Ion leakage (**a**), H_2_O_2_ production (**b**), MDA production (**c**) and cell viability (**d**) of *A. thaliana* leaves treated with CA. (**a**–**c**) Leaves of three-weeks old *A. thaliana* plants, sprayed with a solution containing 10 or 100 μM CA, were collected 6 and 24 hat and REC%, DAB production and MDA content estimated as reported in Section 5.5, Section 5.6 and Section 5.7, respectively. (**d**) Leaves of three-weeks old *A. thaliana* plants, sprayed with a solution containing 10 or 100 μM CA, were collected after 1 and 3 dat and cell viability estimated as reported in Section 5.8. Results from three independent experiments are reported; values are expressed as the mean ± SEM. Statistical significance was attributed by Student’s test (* *p* < 0.05).

To evaluate the kind and extent of cell injury brought about by CA, ion leakage, hydrogen peroxide, and MDA production assays were performed on *A. thaliana* leaves treated with micromolar concentrations of the toxin. Measurements showed that values of relative electric conductivity (REC %), which is an indirect estimate of membrane damage, increased 6 hat and were further augmented 24 hat; moreover, the effect was dependent on CA concentration from 10 to 100 μM (Figure 3a). Among the different ROS species whose production is elicited in plants by abiotic or biotic stress, H_2_O_2_ is the most stable one; it can be detected in situ by oxidation of 3, 3′diaminobenzidine (DAB) chromogen by endogenous peroxidases, to form a brownish precipitate. Figure 3b shows that CA 10 μM increased hydrogen peroxide production as early as 6 hat and that the production was maintained 24 hat. The same trend, but to a higher extent, was observed when 100 μM CA was administered to leaves. MDA is produced from membrane-lipid oxidation by ROS, thereby being an indirect measure of membrane damage. Figure 3c shows that 100 μM CA treatment induced MDA production 6 hat, which was further increased 24 hat. Taken together, these results indicate that CA treatments, particularly at 100 μM concentration, induced oxidative stress and affected membrane integrity. Finally, results from Trypan blue assay (Figure 3d) showed that 100 μM CA significantly reduced (about 30%) cell viability 3 dat, suggesting that membrane insult can lead to irreversible impairment of cell functions.

### 2.3. CA Induced the Transcription of Defense-Related Genes in A. thaliana Leaves

**Figure 4:**
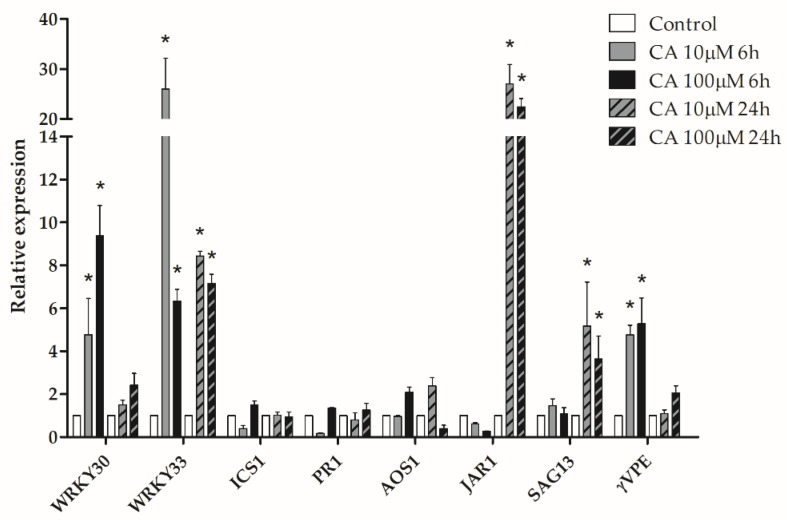
qRT-PCR analysis of defense-related genes transcription in *A. thaliana* leaves treated with CA. Leaves of three-weeks old *A. thaliana* plants were sprayed with a solution containing 10 or 100 μM CA and collected 6 and 24 hat. Total mRNA was extracted and the qRT-PCR analysis of relative expression of *WRKY30, WRKY33, ICS1, PR1, AOS1, JAR1, SAG13* and γVPE genes performed as described in Section 5.11. Results from three independent experiments are reported; values are expressed as the mean ± SEM. Statistical significance was attributed by Student’s test (* *p* < 0.05).

The effect of administration of 10 or 100 μM CA to *A. thaliana* leaves on the transcription of genes involved in the resistance to pathogen stress was tested by qRT-PCR 6 and 24 hat. As shown in Figure 4, CA elicited the transcription of *WRKY33* and *WRKY30* genes. WRKY33 is a key transcriptional regulator of the defense response to necrotrophic fungi, involved in the negative regulation of the salicylic acid (SA) pathway and in the positive regulation of jasmonic acid (JA) pathway [20]. Moreover, in *A. thaliana*, it is an interactor of ATG18a, which determines the activation of autophagy upon infection of the necrotrophic fungus *Botrytis cinerea* [21]. Levels of *WRKY33* transcripts were highly increased by 10 and 100 μM CA, both 6 and 24 hat. Likewise, transcripts of the *WRKY30* gene were also increased upon CA challenge 6 hat but declined 24 hat. WRKY30 is a positive regulator of resistance to necrotrophic fungal pathogens, whose induction is correlated to elevated levels of transcripts of JA biosynthetic genes and accumulation of JA during pathogen challenge [22]. In accordance with *WRKY* gene transcripts up-regulation, the up-regulation of transcripts of the *AOS1* (allene oxide synthase 1) and *JAR1* (jasmonoyl-L-amino acid synthase) JA biosynthetic genes was observed. Conversely, transcripts of the *ICS1* (isochorismate synthase 1) and *PR1* (pathogenesis-related protein 1) genes, involved in the biosynthesis and response to SA, respectively, were not increased by CA treatment. We also tested the transcription of *SAG13* and *γVPE* genes, which regulate PCD in plants, a phenomenon that can be induced by the pathogen to facilitate infection or that can be elicited as a defensive response by the plant immune system. *SAG13,* which is induced by oxidative stress, is considered a marker of senescence-like cell death and is a positive regulator of *A. thaliana* defense response to *Botrytis cinerea* [23]. As shown in Figure 4, 10 and 100 μM CA administration brought about a consistent increase in *SAG13* gene transcripts 24 hat. γVPE is a protease with caspase-1-like activity which triggers the vacuole-mediated cell death, a form of PCD exclusive for plants which occurs from vacuole membrane rupture and release of hydrolytic enzymes into the cytosol [24,25,26]. Vacuolar PCD can be part of the hypersensitive response (HR) of the host against biotrophic pathogens or can be induced by toxins secreted by necrotrophic fungi, in order to kill plant cells and promote pathogen growth [27]. As shown in Figure 4, transcript levels of γ*VPE* were significantly increased by administration of 10 and 100 μM CA 6 hat, to decline 24 hat. On the whole, qRT-PCR results proved at molecular level that CA is a determinant of the pathogenicity of *Seridium* genus fungi, which elicits in the host the transcription of key genes of the plant defense response to necrotrophic fungi, involving hormonal signaling, and PCD.

### 2.4. CA Impaired Plasma Membrane Potential, Mithocondria Functionality and Vacuole Integrity in Protoplasts from A. thaliana Leaves

**Figure 5:**
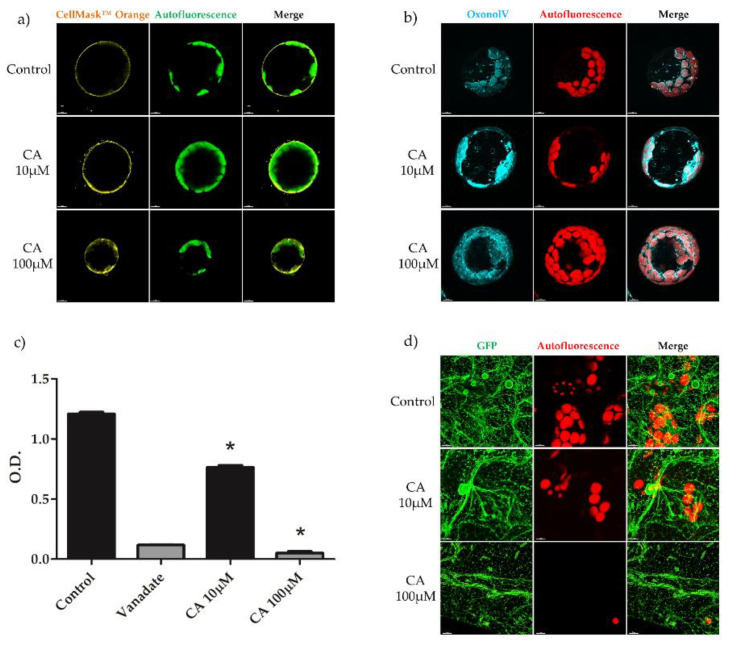
Confocal microscope imaging of plasma membrane (**a**) and plasma membrane potential of protoplasts from *A. thaliana leaves* (**b**). Confocal microscope imaging of the ER network of *A. thaliana* leaves (**d**). ATP-phosphohydrolytic activity of purified plasma membrane vesicles from *A. thaliana* roots (**c**). (**a**,**b**) Protoplasts prepared from leaves of three-weeks old *A. thaliana* plants were treated with 10 or 100 μM CA and then analyzed by confocal microscope, 1 hat. For plasma membrane visualization, 10 µM CellMask Orange^TM^ at 554 nm excitation and 567 nm emission, respectively, was used. To monitor plasma membrane potential, 10 µM Oxonol V at 560 nm excitation and 640 nm emission, respectively, was used. Bar = 7 μM. (**d**) Leaves from three-weeks old, GFP-tmKKXX-expressing *A. thaliana* plants were treated with 10 or 100 μM CA and then analyzed by confocal microscope at 488 nm for GFP excitation and 520 nm for emission, 1 hat. Bar = 7 μM. (**c**) Two-phase partitioned plasma membranes from *A. thaliana* roots were incubated with 20 μM vanadate, 10 or 100 μM CA, for 10 min and then ATP hydrolysis estimated as reported in Section 5.10. Results from three independent experiments are reported; values are expressed as the mean ± SEM. Statistical significance was attributed by Student’s test (* *p* < 0.05).

To ascertain whether and which subcellular organelles could be targeted by CA, confocal microscopy experiments were performed on protoplasts from *A. thaliana* leaves incubated with micromolar concentrations of the toxin. The morphology and/or functionality of subcellular organelles were monitored by means of specific fluorescent dyes. For imaging of plasma membrane, protoplasts were incubated with the fluorescent probe CellMask Orange, which specifically stains plasma membrane. Moreover, plasma membrane potential was monitored by using Oxonol V, which is accumulated inside the cell in dependence of its transmembrane potential. Results, shown in Figure 5a, demonstrated that administration of 10 or 100 μM CA, did not compromise plasma membrane integrity 1 hat. Indeed, in presence of CA, protoplasts maintained their spherical shape and turgor, and chloroplasts in all samples grouped very close to the plasma membrane. On the other hand, Oxonol V was accumulated preferentially in CA-treated protoplasts (Figure 5b). This negatively charged dye accumulates in compartments with an inside-positive potential. Hence, accumulation in CA-treated protoplasts indicates that the toxin depolarized the plasma membrane potential, suggesting that it targets PMA, the master enzyme for the generation and maintenance of the transmembrane potential. This suggestion was verified by investigating the effect of CA on the ATP-phosphohydrolytic activity of purified (two-phase partitioned) plasma membrane preparations. In Figure 5c it is shown that CA strongly inhibited the ATPase activity of plasma membrane vesicles and that the effect was concentration-dependent. In particular, 100 μM CA almost completely inhibited ATP hydrolysis, as done by the PMA specific inhibitor vanadate. Therefore, on the whole, these results demonstrated that CA targeted plasma membrane where it inhibited PMA, leading to transmembrane potential impairment. To visualize the ER structure, leaves from *A. thaliana* plants expressing the fluorescent protein GFP-tmKKXX, which specifically localizes in the ER, were used [28]. Results of confocal microscopy showed that the integrity of the continuous ER network was not significantly affected by 10 or 100 μM CA (Figure 5d) Hence, this result indicated that ER is not a primary target of CA.

**Figure 6:**
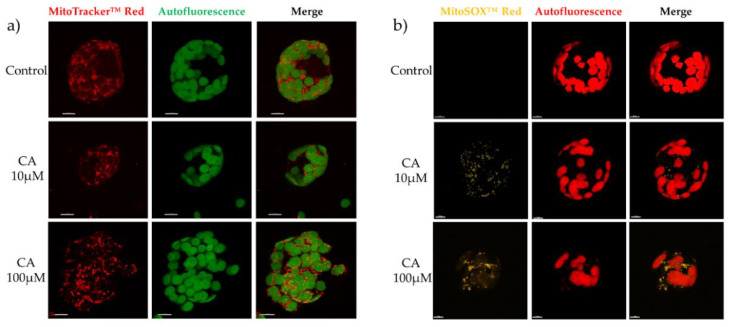
Confocal microscope imaging of mitochondrial network (**a**) and of mitochondrial ROS (**b**) of protoplasts from *A. thaliana* leaves. Protoplasts prepared from leaves of three-weeks old *A. thaliana* plants were treated with 10 or 100 μM CA and then analyzed by confocal microscope, 1 hat. (**a**) For mitochondrial network imaging, 1 µM MitoTracker at 579 nm excitation and 599 nm emission, respectively, was used. To monitor mitochondrial ROS production, 3 µM MitoSOX at 510 nm excitation and 580 nm emission, respectively, was used. Bar = 7 μM.

On the contrary, when protoplasts were incubated with the fluorescent probe MitoTracker Red that accumulates into functional mitochondria in dependence of the membrane potential, or with MitoSOX Red, which becomes highly fluorescent upon oxidation by mitochondrial oxygen superoxides, positive results were obtained. MitoTracker Red staining showed that treatment of protoplasts with 10 μM CA determined a partial fragmentation of the mitochondrial network, which was increased in samples incubated with 100 μM CA, where also clustering of mitochondria was visible (Figure 6a). MitoSOx Red staining showed that fragmentation of the mitochondrial network was paralleled by a corresponding increase of ROS production upon CA incubation from 10 to 100 μM (Figure 6b). These results suggest that CA targeted mitochondria and that the interaction with the mitochondrial membrane hampered its functionality, possibly resulting in depolarization and massive ROS production, which in turn brought about membrane lipid peroxidation and network fragmentation.

**Figure 7:**
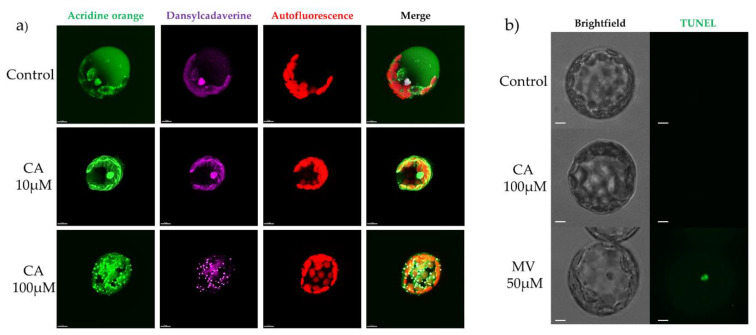
Confocal microscope imaging of vacuoles of protoplasts from *A. thaliana* leaves treated with CA (**a**). Fluorescence microscope imaging of nuclear DNA fragmentation of protoplasts from *A. thaliana* leaves treated with CA (**b**). (**a**) Protoplasts prepared from leaves of three-weeks old *A. thaliana* plants were treated with 10 or 100 μM CA and then analyzed for vacuole imaging with 30 µM acridine orange at 488 nm excitation and 526 nm emission, respectively (green fluorescence), or for autophagosome-like vesicles imaging, with 10 μM MDC at 488 nm excitation and 512 nm emission, respectively (purple fluorescence), 1 hat. Bar = 7 nm. (**b**) Protoplasts from leaves of three-weeks old *A. thaliana* plants were treated with 100 μM CA or 50 μM MV, as a positive inducer of apoptosis-like cell death, and then DNA fragmentation visualized 1 hat, by using the in situ Cell Death Detection Kit from Roche (Sigma-Aldrich, St. Louis, MO, USA) and an optical/epifluorescence microscope.

Incubation with acridine orange, which accumulates in acidic compartments, allowed the analysis of vacuole morphology. As reported in Figure 7a, in control protoplasts a diffuse green fluorescence, corresponding to the cellular volume occupied by intact vacuoles, was observed, whereas upon incubation with 10 or rather 100 μM CA, the dim and diffused green vacuolar fluorescence disappeared and a number of bright green fluorescent puncta became visible. These results indicated that CA treatment determined disgregation of the vacuole, a fact that is reminiscent of vacuolar PCD induction, which is known to occur during necrotrophic fungi infection and consequent plant immune response [25]. Besides the vacuole, acridine orange accumulates in other acid compartments, suggesting that the bright green-fluorescent puncta might be acidic vesicles with lysosomal function, such as autophagosome-like vesicles. This hypothesis was corroborated by the positive results obtained by staining with monodansylcadaverine (MDC) which is considered a marker for autophagosomes [29]. This latter result suggests that upon CA treatment, concomitantly to vacuolar PCD, autophagy was triggered, in order to limit toxin-induced PCD, as part of the immune response contrasting necrotrophic pathogen invasion [30,31]. To rule out the induction of an apoptosis-like PCD, we tested nuclear DNA fragmentation. In fact, DNA cleavage is a hallmark of apoptosis-like PCD in plants, a feature lacking in vacuolar PCD. DNA fragmentation was investigated in *A. thaliana* protoplasts treated with 100 μM CA by TUNEL staining and fluorescence microscopy. Results, reported in Figure 7b, showed that CA treatment, different from that with methyl viologen (MV), did not result in nuclear fluorescence, thereby excluding the occurrence of apoptosis-like PCD.

### 2.5. CA Induced ATG8 Lipidation in A. thaliana Leaves

**Figure 8:**
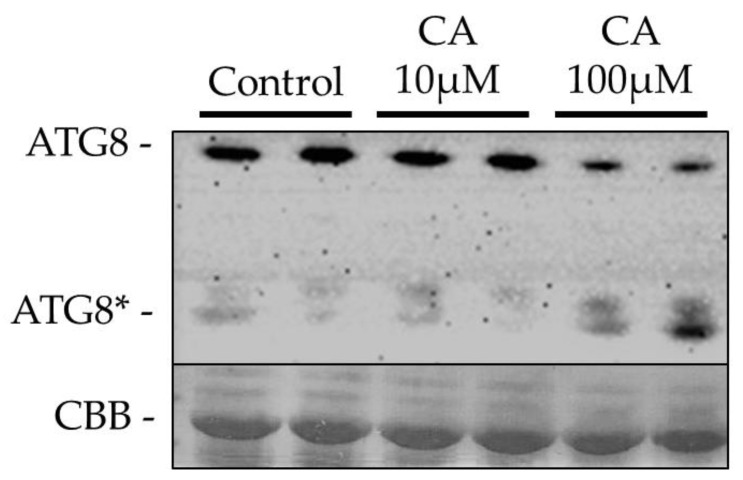
Western blotting with anti-ATG8 antibodies of extracts of *A. thaliana* leaves treated with CA. Leaves from three-weeks old *A. thaliana* plants sprayed with a solution containing 10 or 100 μM CA, were detached 24 hat and soluble proteins extracted under the conditions described in Section 5.13. Proteins were separated by SDS-PAGE onto 16% gels, blotted onto a PVDF membrane and incubated with anti-ATG8 antibodies. Protein bands were revealed by using horseradish-peroxidase conjugated, anti-rabbit secondary antibodies and luminol. 20 μg total protein each sample were loaded. ATG8* = ATG8-PE adduct, resulting from lipidation of ATG8. CBB, Coomassie Brilliant Blue staining.

Autophagy, recycling cellular constituents to preserve cell homeostasis, is up-regulated upon abiotic or biotic stress, in order to extend cell survival [32]. The autophagic process encompasses distinct stages, which are regulated both in animals and plants by many autophagy-related genes (ATG). The ATG8/ATG12 ubiquitin-like complex functions in the last phase of phagophore maturation and involves conjugation of ATG8 to phosphatidylethanolamine (PE), to form the ATG8-PE adduct [33], which is therefore considered a biochemical marker of autophagy. Hence, considering positive results with MDC staining of protoplasts, suggesting the elicitation of autophagy upon CA challenge, we performed western blotting experiments with anti-ATG8 antibodies on *A. thaliana* leaves treated with 10 and 100 μM CA for 24 h, in order to detect the formation of the ATG8-PE adduct. Results reported in Figure 8 showed that upon CA treatment, the SDS PAGE migration profile of ATG8 was changed. In fact, unlike control samples, in CA-treated samples a decrease in the higher molecular weight (MW) band recognized by antibodies was observed, together with an increase in a lower MW band corresponding to the ATG8-PE adduct, which is known to migrate faster in SDS PAGE. Moreover, the extent of the relative variations between the higher and lower MW bands was dependent on the CA concentration. These results, together with confocal imaging data of MDC staining of protoplasts, demonstrated that CA action in the plant cell involves activation of autophagy.

## 3. Discussion

Necrotrophic fungi feed on dead tissues, so that successful infection relies on their capacity to overcome the plant defense response, in order to kill cells. For this purpose, necrotophs produce a wide array of toxic metabolites, phytotoxins or other effectors, which help to establish the disease [34]. Non-specific phytotoxins are the most widespread, since they are produced by broad-host-range fungi that, infecting a wide range of plant species, are among the major causes of annual crop loss worldwide. Hence, deepening the knowledge about the mode of action of phytotoxins is not only of scientific but also of practical interest for a better management of plant diseases as well as exploitation of their diverse biological properties. Nevertheless, molecular information for most phytotoxins is still lacking or largely incomplete. In this study, we investigated the mechanism(s) of phytotoxicity of CA, one of the major metabolites of the *Seiridium* genus fungi, causal agents of the canker disease of several plant species of the Cupressaceae family. CA, administered at micromolar concentrations to *A. thaliana* plants, reduced growth and produced chlorotic areas in leaves, whereas when the toxin was supplemented into the growth medium, it strongly inhibited root proton extrusion. These toxic effects were correlated to chlorophyll loss, ion leakage, MDA and H_2_O_2_ production, thereby strongly suggesting that CA brings about in plant cells membrane insult and ROS overproduction. Remarkably, in vivo inhibition of root proton extrusion was paralleled by in vitro inhibition of the ATP-phosphohydrolytic activity of purified plasma membrane vesicles, thereby indicating that the toxin targets PMA, the master enzyme for ion transport and growth. Early cellular responses to pathogen infection take place at the plasma membrane and PMA can be the target of multiple pathogens and elicitors during infection. One of the earliest events of the plant immune response is modulation of the extracellular pH, and various elicitors induce extracellular alkalinization [35]. Toxin administration to leaves determined also the induction of defense-related genes. In fact, CA: (i) up-regulated *WRKY33* and *WRKY30*, key transcriptional regulators of the immune response to necrotrophic fungi; (ii) down-regulated *ICS1* and *PR-1*, involved in the synthesis and response to SA, respectively; (iii) up-regulated *AOS1* and *JAR1* involved in JA biosynthesis; (iv) up-regulated *SAG13* and *γVPE*, involved in the induction of senescence and vacuole-mediated PCD, respectively. qRT-PCR results demonstrated that CA is a virulence factor, able in itself to trigger the plant defense response, which is regulated by typical elements of immunity to necrotrophic fungi, such as JA and WRKI factors and that ultimately leads to cell death induction. Confocal microscopy experiments on protoplasts incubated with organelle-specific fluorescent dyes allowed the identification of subcellular structures affected by the toxin. Results demonstrated that CA: (i) depolarized the plasma membrane potential, a fact that together with inhibition of root proton extrusion and of PMA activity, definitely demonstrates that the proton pump of the plasma membrane is a target of the toxin; (ii) disrupted the mitochondrial network and impaired transmembrane potential, bringing about ROS overproduction; (iii) determined vacuole disruption and induced the formation of acidic, autophagosome-like vesicles.

Taken together, our results are consistent with a model according to which CA affects different membrane structures in the plant cells, impairing their functionality and inducing cell death. CA directly targets and inhibits PMA, determining transmembrane potential depolarization and, possibly, extracellular alkalinization, events that are usually linked during pathogen infection to Ca^++^ influx, oxidative burst and cell death [35]. Recently, it has been shown in *Nicotiana benthamiana* that the N-terminal domain of a pepper nucleotide-binding leucine-rich (NRL) immune receptor associates to PMA, bringing about depolarization and inducing cell death [36]. CA affects mitochondria functionality, hampering transmembrane potential, and bringing about mitochondrial ROS overproduction, which in turn also leads to PCD induction. A growing body of evidence supports the idea that a common strategy of necrotrophic pathogens is to subvert the plant defense response, exploiting the plant oxidative burst and consequent PCD elicitation, to derive nutrition from dead tissues [34]. Accumulating evidence suggests also that fungal phytotoxins play a role in the manipulation of the plant immune response and in the induction of PCD [37]. In fact, AAL toxin, secreted by the tomato pathogen *Alternaria alternata* and fumonisin B1 (FB1) produced by the maize pathogen *Fusarium moniliforme*, induce apoptosis-like PCD in tomato [38], and botrydial, a phytotoxic sesquiterpene of *Botrytis cinerea* induces the hypersensitive response (HR) in *A. thaliana*, together with expression of SA- and JA-dependent defensive genes [39]. Membranes’ depolarization and oxidative burst (even though a direct effect of CA on the tonoplast cannot be ruled out) determine vacuole disgregation. This event, together with *γVPE* gene up-regulation, demonstrates that CA triggers vacuole-mediated PCD. In this process, γVPE, a vacuole-located enzyme with caspase 1-like activity, plays a pivotal role, provoking vacuole rupture and initiating the proteolytic cascade, which leads to cell death [24,25,26]. Interestingly, it has been shown that the fungal toxin FB1 is able, per se, in the absence of the pathogen, to bring about in *A. thaliana* vacuolar PCD, which requires γVPE induction [27].

Remarkably, tonoplast disgregation is accompanied by formation of MDC-accumulating autophagosome-like vescicles, a finding that together with induction of ATG8 lipidation, demonstrates that CA challenge leads also to activation of autophagy. The autophagic process is a major catabolic pathway which plays a fundamental role in the regulation of development, as well as stress-associated, cell death [40]. Although it is still a matter of debate whether autophagy plays a pro-death or pro-survival role in controlling PCD, and originally the formation of autophagosomes in dying cells has led to the belief that autophagy is necessary to the execution of cell death, mounting evidence supports the idea that in the plant immune response to necrotrophs, autophagy acts as a pro-survival mechanism, removing detrimental materials in the cell, including ROS and damaged organelles, thereby mitigating the death process exploited by the pathogen to kill cells and derive nutrients [21,41]. Hence, our finding provides for the first time evidence of the ability of a phytotoxin to elicit autophagy in the plant cell and corroborates the hypothesis of a mitigating function of autophagy in the regulation of cell death associated to basal immunity against necrotrophic fungi. However, for the definitive confirmation of the pro-survival role of the autophagic process elicited by CA, further information is necessary. It can be obtained by investigating the response to the toxin of *A. thaliana* autophagic mutants. This will be the object of future work.

## 4. Conclusions

CA, a phytotoxin produced by *Seiridium* genus fungal pathogens, induced in *A. thaliana* tissues ion leakage, hydrogen peroxide and MDA production, plasma membrane H^+^-ATPase inhibition and elicitation of key genes of the plant defense response to necrotrophic fungal pathogens, including WRKY transcription factors, JA biosynthesis genes as well as vacuolar PCD- and autophagy-related genes. In *A.thaliana* protoplasts, CA hampered plasma membrane potential, brought about mitochondria network disruption and ROS overproduction as well as vacuole disgregation, together with PCD and autophagy induction.

## 5. Materials and Methods

### 5.1. Cyclopadic Acid, Plant Material, Growth Conditions and Treatments

Cyclopaldic acid was obtained by crystallization from *Diplodia cupressi* (syn. *Seiridium cupressi*) culture filtrates, as previously described [7]. Briefly, the fungal culture filtrate was acidified with HCl to pH 4 and exhaustively extracted with EtOAc. The organic extract was dried, filtered and evaporated under reduced pressure, yielding an oily residue. This residue was washed with CHCl_3_ and purified by column chromatography and crystallized from MeOH/CHCl_3_ 1:1 *v*/*v* and then from H_2_O. The crystals obtained had the same physical and spectroscopic properties as previously reported [7]. The *D. cupressi* strain was isolated from infected cypress plants in Kos, Greece as previously reported [7], and deposited in the collection of the “Dipartimento di Agraria, Sezione di Patologia Vegetale ed Entomologia, Università di Sassari” Sassari, Italy. For plant growth, 40 mg of seeds from wild type (WT), Columbia 0 (Col-0) ecotype, *A. thaliana* plants, or from GFP-tmKKXX-expressing *A. thaliana* plants (provided by Prof. P. Schäfer, School of Life Sciences, Warwick University, Coventry, UK), were added to 1 mL of deionized water, and kept at 4 °C for 4 days, in the dark. After stratification, seeds were suspended in 0.1% agarose and scattered in pots filled with universal soil. The pots were placed in a climatic chamber (VB1514 Vötsch, Rosenfeld, Germany), at 22 °C and 80% humidity, with a 16/8 h light/dark cycle, and the seeds kept growing for 3 weeks. For CA treatments, three weeks-old plants were sprayed with 10 or 100 μM solutions of the toxin, containing 0.05% Tween 20, until a complete wetting was observed. For growth inhibition experiments, plants were grown in vitro in MS medium, containing or not 10 or 100 μM CA, for seven days.

### 5.2. Preparation of Protoplasts

Protoplasts’ purification was performed as described by [42]. Twenty leaves of three weeks-old *A. thaliana* plants were cut into 0.5–1 mm stripes, submerged into the enzyme solution (1% cellulase R-10, 0.25% macerozyme R-10, 0.4 M mannitol, 20 mM KCl, 20 mM MES-OH, 10 mM CaCl_2_, pH 5.7), incubated 30 min under vacuum, and then 150 min, in the dark at 25 °C. After digestion the mixture was filtered with 100 μM cell strainer (Falcon REF352340, Corning, Deeside, UK) and centrifuged at 100× *g* for 2 min. The protoplast pellet was washed twice with 5 mL of ice-cold 2 mM MES-KOH buffer pH 5.7, containing 154 mM NaCl, 125 mM CaCl_2_ and 5 mM KCl, and resuspended in 3 mL of 2 mM MES-KOH buffer, pH 5.7, containing 0.4 M mannitol and 15 mM MgCl_2_. The number of purified protoplasts was determined with a Thoma cell counting chamber. Purified protoplasts were utilized for confocal microscopy analysis, as reported in Section 5.8.

### 5.3. Total Chlorophyll Assay

The chlorophyll content of *A. thaliana* leaves was estimated as described previously [28]. One hundred mg of control or CA-treated leaves were collected in a Falcon tube with 5 mL of DMSO. After incubation at 65 °C for 90 min and subsequent cooling at 25 °C, the sample was centrifuged at 3000× *g* for 5 min, the supernatant recovered and the chlorophyll content estimated spectrophotometrically, by measuring absorption at 663 nm and 645 nm.

### 5.4. Root Acidification Assay

Root acidification assay was performed according to [43]. For each sample, 30 *A. thaliana* seeds were germinated in Petri dishes in a half-strength MS medium at pH 6, containing 0.03% Bromocresol purple. After two weeks, the seedlings were treated with 10 µM or 100 µM CA. The pH change was monitored by observing the color change of the medium, after 24 h of treatment.

### 5.5. Ion Leakage Assay

Ion leakage assay was performed as described previously [28]. Two hundred mg of leaves from three-weeks old *A. thaliana* plants untreated or treated with 10 µM or 100 µM CA, for 6 h or 24 h, were cut into 5 mm strips, and submerged in 30 mL of deionized water, for 2 h, at 25 °C. After incubation, the electrical conductivity was measured by a conductimeter; this parameter was reported as relative electrical conductivity (REC %) values. Boiled samples were used to determine maximum percentage of electrolyte leakage, which was calculated using the following formula: REC % = C1/C2 × 100 (C1= conductivity at 25 °C; C2 = conductivity at 100 °C).

### 5.6. H_2_O_2_ Production Assay

H_2_O_2_ was detected in leaves from three-weeks old *A. thaliana* plants untreated or treated with 10 µM or 100 µM CA, for 6 h or 24 h, by staining with 3,3′-diaminobenzidine tetrahydrochloride (DAB), as described previously [28]. For each sample, five leaves were submerged in a solution of 10 mM DAB, pH 6.8, containing 0.05% (*w/v*) Tween 20. After vacuum infiltration for 15 min and incubation for 5 min under stirring, leaves were submerged in a bleaching solution of ethanol:acetic acid:glycerol 3:1:1 (**v*/*v*/v*), and boiled for 15 min, in order to remove chlorophyll. After cooling at 25 °C, the bleaching solution was eliminated, fresh bleaching solution added and leaves mounted on glass slides for optical microscopy observation. Quantitative analysis of pixels from leaf images was performed by ImageJ 1.51j8 software.

### 5.7. Membrane-Lipid Peroxidation Assay

Membrane-lipid peroxidation was estimated in leaves from three-weeks old *A. thaliana* plants untreated or treated with 10 µM or 100 µM CA for 6 h or 24 h, by the MDA method, as described previously [28]. One hundred mg of control or CA-treated *A. thaliana* leaves were homogenized in liquid N_2_, suspended in 500 μL of 0.1% trichloroacetic acid (TCA) and centrifuged at 15,000× *g*, for 10 min, at 4 °C. One hundred µL of the supernatant were added to 1.5 mL of 0.5% thiobarbituric acid in 20% TCA, and incubated for 25 min, at 95 °C. After incubation, the reaction was blocked by placing the samples in ice. After cooling at 25 °C, sample absorbance was measured at 532 nm and 600 nm.

### 5.8. Cell Viability Assay

Cell viability was estimated in leaves from three-weeks old *A. thaliana* plants untreated or treated with 10 µM or 100 µM CA, for 1 or 3 days as reported by [44], with modifications. One hundred mg of control or CA-treated leaves were collected in a Falcon tube containing a 0.025% Trypan blue solution. After incubation for 15′ at room T, leaves were washed in deionized H_2_O and submerged in an extraction solution (50% MeOH and 1% SDS) and heated at 60 °C for 30 min. Supernatants were collected and their absorbance measured at 600 nm.

### 5.9. TUNEL Assay

Nuclear DNA nicks, formed as result of apoptotic PCD initiation in *A. thaliana* protoplasts treated with 100 μM CA or with 50 μM methyl viologen for 1 h, were visualized by using the in situ Cell Death Detection Kit, Fluorescein from Roche (Sigma-Aldrich, St. Louis, MO, USA) [45] and an optical/epifluorescence microscope (ECLIPSE TE 2000-E, Nikon, Melville, NY, USA).

### 5.10. ATP Phosphohydrolytic Activity of Plasma Membrane H^+^-ATPase

The ATP-phosphohydrolytic activity of two-phase partitioned plasma membrane vesicles from *A. thaliana* roots was assayed as reported by [46]. Experiments were carried out by using 20 μg plasma membranes for each sample. Treated samples were pre-incubated with 10 or 100 μM CA, or 20 μM vanadate for 10 min at room T.

### 5.11. qRT-PCR Analysis of Genes Expression

For qRT-PCR analysis of gene expression, *A. thaliana* plants untreated or treated with 10 µM or 100 µM CA, were subjected to harvesting of leaves 6 or 24 h after toxin administration. Total RNA was extracted from 100 mg of homogenized leaves using RiboZOL (vWR, Radnor, PA, USA). For cDNA synthesis, 20 μg of total RNA was retro-transcribed by using the FastGene Scriptase II cDNA kit (Nippon Genetics Europe, Düren, Germany), according to the manufacturer’s instructions, and stored at −80 °C until use. qRT-PCR experiments were performed according to [47], using the LightCycler apparatus (Roche, Basel, Switzerland) and the SYBR GREEN dye (PCR Biosystems, London, UK). The 2^−ΔΔCt^ method was applied to evaluate the level of gene expression, using the actin-8 *A. thaliana* gene (*ACT8*) as housekeeping gene. Results represent mean values ± SD of independent experiments (n = 3). Samples were run in technical triplicates. Statistic significance was attributed by Student’s test (*p* < 0.05). The primers used for amplification are listed in Supplementary Table S1.

### 5.12. Confocal Microscopy

Confocal microscopy experiments were performed according to [28], by using protoplasts prepared from *A. thaliana* leaves, as reported in 5.2. In all experiments protoplasts were incubated with 10 µM or 100 µM CA for 1 h before fluorescent dyes staining and confocal microscopy observation. For plasma membrane visualization, 10 µM CellMask Orange^TM^ (Thermo-Fisher) at 554 nm excitation and 567 nm emission wavelengths, respectively, was used. To monitor plasma membrane potential 10 µM OxonolV (Abcam, Cambridge, UK) at 560 excitation and 640 emission wavelengths, respectively, was used. For mitochondria imaging, 1 µM MitoTracker™ Red CMXRos (Thermo-Fisher Scientific, Waltham, MA, USA) at 579 excitation and 599 emission wavelengths, respectively, was used. To monitor mitochondrial ROS production, 3 µM MitoSOX^TM^ Red (Thermo-Fisher Scientific, Cambridge, UK) at 510 nm excitation and 580 nm emission wavelengths, respectively, was used. For vacuole visualization 30 µM Acridine Orange (Sigma-Aldrich, St. Louis, MO, USA) at 488 nm excitation and 526 nm emission wavelengths, respectively, was used. For autophagosome-like vesicles imaging, 10 µM MDC, at 488 nm excitation and 512 nm emission wavelengths, respectively, was used. Images were acquired with a laser-scanning confocal microscope FV1000, Olympus (Hamburg, Germany), using a 60× oil objective (N.A.: 1.35) in z stack mode (step size 0.45 μM). Images were processed by Imaris 6.2.1 software (Bitplane, Zurich, Switzerland).

### 5.13. Protein Extraction, SDS-PAGE and Immunoblotting

One gram of *A. thaliana* leaves untreated or treated with 10 µM or 100 µM CA, for 24 h, was ground in liquid nitrogen in a mortar with pestle. The resulting powder was suspended in 2.5 mL of methanol and left in incubation at 4°C, overnight. After incubation, the sample was centrifuged at 2000× *g*, at 4 °C, for 10 min and the supernatant removed. The pellet was washed three times with ice-cold acetone, and dried under reduced pressure. For SDS-PAGE, 10 mg of the dried pellet were resuspended in 500 μL of 250 mM Tris-HCl pH 6.8, with 2% SDS and sonicated. After sonication, the supernatant was recovered, protein concentration determined and used for SDS-PAGE. Twenty μg samples were subjected to SDS-PAGE as described previously [48] onto 16% gels, using a Mini Protean apparatus (Bio-Rad, Hercules, CA, USA). For immunoblotting, proteins separated by SDS-PAGE were transferred onto a PVDF (polyvinylidene difluoride) membrane equilibrated in Transfer Buffer (39 mM glycine, 48 mM TRIS, 0.1% SDS, 10% methanol, pH 6.8), using a Trans Blot semi-dry Transfer Cell (Bio-Rad, Hercules, CA, USA). After transfer, the membrane was incubated 1h in a blocking solution of 5% no-fatty acid dry milk dissolved in TTBS (20 mM Tris-HCl, 150 mM NaCl, 0.05% Tween, pH 7.5). Then the membrane was incubated with 1:2000 anti ATG8 antibodies from Agrisera (Vännäs, Sweden), overnight, at 4 °C. After incubation, the membrane was washed three times with TTBS and incubated 1h with 1:20,000 horseradish peroxidase-conjugated anti-rabbit secondary antibodies (Bio-Rad, Hercules, CA, USA). Antigen-antibody interaction was revealed with a solution of 1:1 luminol Peroxide Buffer (Euroclone, Milan, Italy). The chemiluminescence signal was acquired with VersaDoc™ 4000 MP (Bio-Rad, Hercules, CA, USA).

### 5.14. Statistical Analyses

Experiments were repeated three times, and the data are expressed as the mean ± standard error of the mean (SEM). GraphPad Prism software 7 (GraphPad Software, Inc., San Diego, CA, USA) was used to test the significance of the data by unpaired *t*-Student’s test; *p* < 0.05 was used to indicate a statistically significant difference.

## Data Availability

Not applicable.

## Supplementary Materials

The following supporting information can be downloaded at: https://www.mdpi.com/article/10.3390/toxins14070474/s1, Table S1: List of oligonucleotides used in qRT-PCR analysis of defense-related gene expression of *A. thaliana* leaves treated with CA.

## Abbreviations

ATG8-autophagy-related gene 8; CA-cyclopaldic acid; dat-days after treatment; hat-hours after treatment; ER-endoplasmic reticulum; GFP-green-fluorescent protein; JA-jasmonic acid; MDA-malondialdehyde; MDC-monodansylcadaverine; MV-methyl viologen; MW-molecular weight; PE-phosphatidylethanolamine; PCD-programmed cell death; PMA-plasma membrane; PVDF-polyvinylidene difluoride; H^+^-ATPase; REC-relative electric conductivity; ROS-reactive oxygen species; SA-salicylic acid; TCA-trichloroacetic acid; TUNEL-terminal deoxynucleotidyl transferase-mediated dUTP nick-end labeling.
