# Supplementary material for: Cyclopaldic Acid, the Main Phytotoxic Metabolite of Diplodia cupressi, Induces Programmed Cell Death and Autophagy in Arabidopsis thaliana
Source: Toxins (Basel). 2022 Jul 11;14(7):474. doi: 10.3390/toxins14070474 (PMC9325063; doi:10.3390/toxins14070474)
Supplement: Supplementary file 1 [file toxins-14-00474-s001.zip › toxins-1783997-supplementary.pdf]

toxins

MDPI

Article

Cyclopaldic Acid, the Main Phytotoxic Metabolite of *Diplodia cupressi*, Induces Programmed Cell Death and Autophagy in *Arabidopsis thaliana*

Simone Samperna, Marco Masi, Maurizio Vurro, Antonio Evidente and Mauro Marra

Table S1. List of oligonucleotides used in qRT-PCR analysis of defense-related gene expression of *A. thaliana leaves* treated with CA.

Gene	Primer sense 5′-3′	Primer antisense 5′-3′
WRKY30	GAAGCCCCAAGAGCGATGAT	CTGAATCCATCGTCCAGCGT
WRKY33	CTCGTGGTAGCGGTTACGCC	CCTTTGCTCTAGAGAATCCACC
ICS1	CTCTCCAACTCCATTCACTA	TCTCCATCACAACCATTCA
PR1	CGAGAAGGCTAACTACAACTACG	ACACCTCACTTTGGCACATC
AOS1	ATAACTGCGTGGTCTGAA	ATAACTGCGTGGTCTGAA
JAR1	AACTGTGGTCTCAATGGAA	GTAAGGCTCTAATTCAACATCA
SAG13	GGGCTTGGGAGAGAACTCAA	TGGCTAGTTCCTCCACCAC
γVPE	AGTGGGAAGGTTGTGGATAG	CTCCAGGGCAATAGGTACC
